# Optimization scheduling of microgrid comprehensive demand response load considering user satisfaction

**DOI:** 10.1038/s41598-024-66492-1

**Published:** 2024-07-11

**Authors:** Chaoliang Wang, Xiong Li

**Affiliations:** State Grid Zhejiang Marketing Service Centre, Hangzhou, Zhejiang China

**Keywords:** Micropower grid, Demand response, Flexible load control, User satisfaction, Energy science and technology, Engineering

## Abstract

The original load control model of microgrid based on demand response lacks the factors of incentive demand response, the overall satisfaction of users is low, the degree of demand response is low, the Time Of Use (TOU) price of peak-valley filling capacity is weak, and the peak-valley difference of load curve is large. Regarding the limitations of the current microgrid demand response model, this study further optimizes the flexible load control strategy and proposes a two-objective optimization model based on price and incentive. Meanwhile, the model is solved using an improved chaotic particle group algorithm. Finally, the microgrid load data were selected for simulation analysis. The simulation results showed that the comprehensive demand response of flexible control model proposed increased the overall satisfaction of users by 9.51%, the overall operating cost of microgrid suppliers decreased by 12.975/ten thousand yuan, the peak valley difference decreased by 4.61%, and the user demand response increased by 27.24%. The model effectively improves the overall profit of the supply side of the microgrid, improves the user satisfaction, and maximizes the linkage benefits of the supply and demand of the micro grid. In addition, the model effectively reduces the phenomenon of distributed power supply in the microgrid, and realizes the supply and demand matching of the whole load in the microgrid.

## Introduction

**Figure 1:**
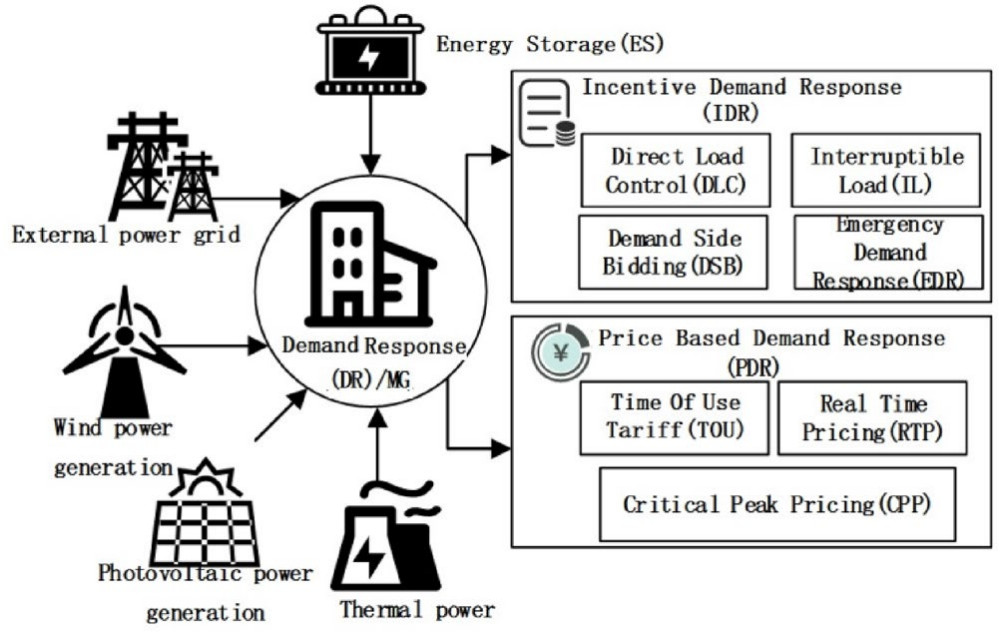
The demand response classification diagram.

To improve the use efficiency of power resources and meet the demand of many new energies access, the distribution grid has gradually developed towards intelligent and integrated development. User-side demand response technology and bidirectional optimization of power have become the focus of load control in the power market^1,2^. During the development of smart grid, demand response has changed (Fig. 1), among which, the role of end users on the demand side has changed significantly^3,4^. Demand response technology changes the original electricity behavior and habits through electricity price adjustment and incentive policy, guides users to actively participate in the power optimization scheduling, and realizes the flexible load control^5,6^. In this process, users use generalized demand response resources to optimize their own electricity behavior to improve electricity economy^7,8^. Power grid enterprises guide user electricity behavior through flexible time-sharing electricity price, and achieve a two-way game with users, thus maintaining the balance of power supply and demand^9,10^. Before the development of demand response, when the contradiction between supply and demand of power was prominent, orderly power consumption and load control were mainly relied on to reduce the peak power load. The power demand response is a means of “market means-intelligent technology-internet”, which gives users more choices through market means and optimizes the balance between power supply and power demand. Therefore, it is more flexible and efficient. Mohammad integrated user demand scheduling, and established a flexible residential user model with electric vehicles and high pressure load to alleviate the congestion of the distribution network at peak load^11^. In order to improve the problem of energy distribution shortage in smart micro-grid, Garcia reduced load demand based on demand response constraints, optimized resource scheduling and increased energy consumption of micro-grid under the premise of ensuring the safe operation of grid^12^. Bishwajit Dey evaluated various fitness functions, including those considering different grid pricing and grid participation strategies for economic scheduling^13^. They combined the Crow Search Algorithm (CSA) with the Arithmetic Optimization Algorithm (AOA) for mathematical modeling, achieving notably good optimization results. Srikant Misra proposed a Demand Side Management (DSM) method based on hybrid intelligent technology to strike a balance between minimizing generation costs and reducing pollutants from distributed energy sources in low-voltage grid-connected microgrid systems^14^. Tapas Chhualsingh researched the feasibility of low-voltage microgrid systems, employing a novel, robust, mixed swarm intelligence optimization algorithm as a research optimization tool, with results showing effective peak reduction^15^. Bishwajit Dey designed a new intelligent algorithm to maximize cost reduction in microgrid systems overall^16^. Bo Xu introduced a hybrid demand response mechanism combining real-time pricing and incentives to implement a demand response scheme for grid operator scheduling^17^. Weiqi Meng proposed an optimization strategy based on both temporal and spatial domains, integrating the hybrid demand response mechanism into scheduling to ensure the interests of all participants^18^. Compared with the swarm evolution optimization algorithm, Chaos Particle Swarm Optimization (CPSO) has enhanced the global search ability, and can effectively enhance the diversity of particle swarm by introducing randomness and ergodicity in chaos theory, so as to avoid premature convergence of the algorithm to the local optimal solution. The use of chaotic mapping or chaotic sequence makes the exploration of search space more extensive, which helps to jump out of the local optimal and find the global optimal solution. In addition, the CPSO algorithm is especially suitable for the optimization problems with highly nonlinear and complex constraints due to its inherent characteristics, which can effectively reveal the global structure of the problem and find high-quality solutions. The recently developed swarm evolutionary optimization algorithms have good parallelism and robustness, but may fall into local optimality when dealing with some high-dimensional complex problems. The CPSO algorithm is helpful to escape local optimization and enhance global search ability.

However, the user's willingness to use electricity is disturbed by multiple factors such as electricity bill and his own consumption elasticity. The user load curve is no longer the traditional typical load mode, and it is more difficult for load control^19^. Therefore, to reduce the investment cost of power supply more effectively, it is particularly important to study the demand response behavior characterization of power users. Among them, the power grid company is the manager and leader of the power industry, which can realize the supervision of the whole process of power production to consumption. Therefore, power grid companies have rich data resources. This paper mainly through docking with power grid enterprises, the typical intra-day user load survey and data analysis, to obtain the user electricity curve.

**Figure 2:**
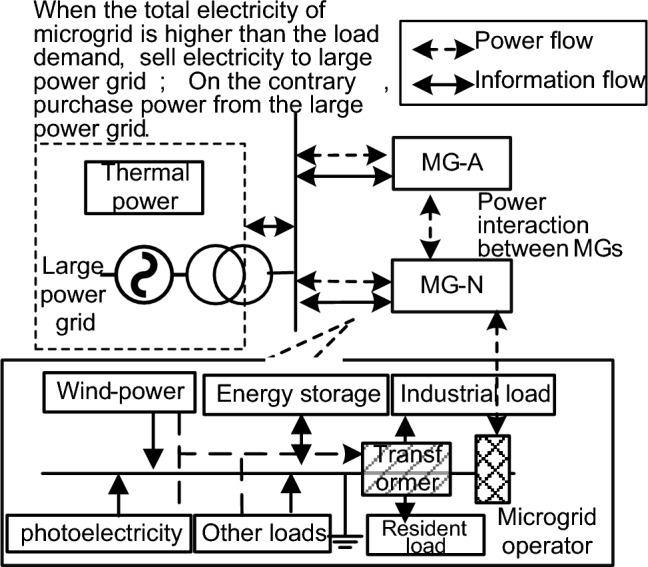
The overall framework of microgrid.

**Figure 3:**
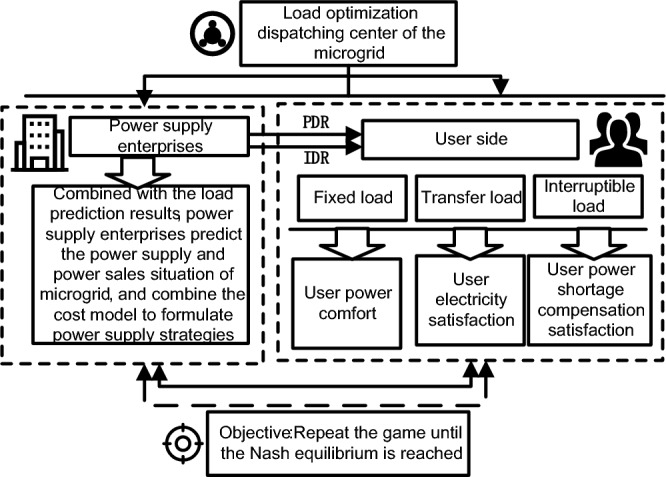
The overall framework diagram of microgrid load optimal dispatching considering comprehensive demand response.

Different from the conventional power grid structure, microgrid is an autonomous power grid with both power generation, transmission, and distribution. The overall framework of microgrid is shown in Fig. 2. The rise of micro-grid in power system has brought new challenges and opportunities to demand response technology^20,21^. Although experts and scholars at home and abroad are relatively mature for demand response research, but there are still the following problems^22–24^: the existing microgrid load prediction and load control process is mostly focused on the impact of time-sharing electricity price on load control, ignoring the incentive and price parallel comprehensive demand response on microgrid load control, leading to the serious phenomenon of microgrid wind and light. Therefore, according to the impact of the current demand response on the user satisfaction and the overall benefit of the microgrid, the article only focuses on the limitations of the time-sharing electricity price, further optimizes the flexible control strategy of the microgrid load, and puts forward a two-objective optimization model based on the comprehensive demand response of the price type and the incentive type. Meanwhile, the model is solved by combining the chaotic ideas and the Particle Swarm Optimization (PSO) algorithm using an improved chaotic particle group algorithm. Finally, the load data of micro grid in a certain region was selected for simulation and analysis to maximize the linkage benefits of supply and demand of micro grid. The overall framework diagram of microgrid load optimal dispatching considering comprehensive demand response is shown in Fig. 3.

## Methodology

### Model of wind power, photovoltaic and energy storage output in microgrid

With the continuous development of human society and economy, the consumption of electricity energy continues to increase, which leads to the gradual increase of energy consumption and the increasing shortage of resources. In order to reduce the use of fossil fuels, countries around the world have been focusing on the use of ecological resources to generate electricity, with remarkable results. In recent years, Wind Power (WP) generation and Series–Parallel (SP) interconnected Photovoltaic (PV) power generation have entered a golden age of development, with power generation costs greatly reduced and equipment production capacity continuously improved^25,26^. The improved quality of power generation equipment, such as solar panels, frequency conversion governors and fans, has made wind power and photovoltaic power an increasing proportion of total power generation, further reducing mankind's dependence on traditional energy sources. At the same time, it also reduces the emission of harmful gases to a large extent^27,28^. Micro-grid contains many types of micro-power. The article mainly considers WP and PV. Different types of micro-power have different output characteristics. At the same time, in order to give full play to the advantages of clean resources in energy saving and emission reduction, and to hedge against the randomness, volatility and intermittent defects of wind and solar energy, a certain amount of Distributed Energy Storage (DES) equipment will be added in the microgrid^29,30^. They can store power in case of power generation redundancy and transmit power in case of power shortage, to avoid power waste. The energy storage system in microgrid mainly uses five kinds of energy storage technologies, which are battery, superconducting magnetic energy, liquid flow battery, super capacitor and flywheel. DES store electricity during off-peak periods, discharge it during peak periods, and provide security when the power supply is interrupted. Distributed energy storage will become an important part of the future power system and become the main regulation means and security elements of smart grid. The author provides definitions for various terms related to power generation.

(1) The active power output model can be described as follows^31^:

1 $$ P_{wp} = \frac{1}{2}\rho C_{p} \left( {\alpha ,\beta } \right)\pi R^{2} v^{3} \lambda_{w} $$

In the above equation, ${P}_{wp}$ represents the active power generated by the wind turbine, ${\lambda }_{w}$ represents the ratio of active power generated by the power producing system, $\rho$ denotes the density of the air, ${C}_{p}$ denotes the wind turbine's wind energy usage rate, $v$ A denotes the velocity of the wind, $\alpha$ represents the pitch angle, $\beta$ represents the tip speed ratio.

(2) The following is the model for photovoltaic power generation's (PV) active power output^31^:

2 $$ P_{sp} = IV\lambda_{S} = \left[ {I_{S} - I_{0} \left\{ {\exp \left( {\frac{q}{nkT}(V)} \right)} \right.} \right.\left. {\left. { - 1 \pm \Delta I} \right\}} \right]V\lambda_{S} $$

In the above formula, ${P}_{sp}$ is the active power of the photoelectric system unit, ${\lambda }_{s}$ is the proportion of solar active power consumption, ${I}_{s}$ is the photocurrent, ${I}_{0}$ is the reverse saturation current, $\Delta I$ is the current correction value after the change of the sunlight intensity and heat, $V$ is the output voltage.

(3) The following equation describes the distributed Energy Storage (ES) connected to the distribution network's active power output model^32^:

3 $$ \left\{ {\begin{array}{*{20}l} {P_{stp} \left( t \right) = \eta_{c/dc} P_{n}^{es} \sum\nolimits_{n = 1}^{{G_{n} \left( t \right)}} {state_{n} \left( t \right)} } \hfill \\ {SOC\left( {t_{0} + \Delta t} \right) = SOC\left( {t_{0} } \right) + x\left( {t_{0} + \Delta t} \right)\int_{{t_{0} }}^{t} {\frac{{P_{n}^{es} \eta }}{{E_{es} U_{es} }}} } \hfill \\ \end{array} } \right. $$

In the given equation, represents the efficiency of energy storage equipment during the charging process, whereas represents the efficiency during the discharge process. The variable, represents the active capacity, and represents the running condition for a specific period.

### User demand response model in microgrid

The microgrid adapts its demand response approach based on Time Of Use (TOU) rates, user requests the loading demand and operational statistics of the microgrid. The fundamental concept of micro-grids participating in demand response is to completely integrate and utilize renewable energy sources. Demand response refers to the response service made by the power grid management side according to the users. That is to say, effective coordination of users' demands should be made according to the actual situation of the power grid, so as to ensure the stability of the power grid and users' demands. Furthermore, once the network microgrid users have fully adjusted, there are fluctuations in the power load over time and during specific time periods. The microgrid purchases electricity from the main power grid in order to reduce electricity expenses, while increasing its own use of fresh energy via Distributed Energy Generation (DEG).

(1) After the Demand Response (DR), micro output is less than load demand *P*_*DEG*_ ≤ *P*_*load*_. *MG/large power grid* joint power supply refers to the procurement of electricity from a centralized and extensive power grid. During peak power hours *Tf*, there is a joint power supply from the *external network/MG*, and ES. During the flat peak period *Tp*, there is a joint power supply from the *external network* and *MG*, without any power storage. There is a combined power supply from the *external network/MG* and energy storage charging *x*(*t*) > 0 during the power trough period *Tv*. The incentive policy is primarily manifested in the decreased power purchase cost for internal users and the compensatory profit derived from interruptible load, both of which are influenced by the DR.

4 $$ P_{load,i}^{*} = P_{load,i} \times \left( {\varepsilon_{ii} \frac{{\left( {\pi_{i}^{*} - \pi_{i} } \right)}}{{\pi_{i} }} + 1 + \sum\limits_{\begin{subarray}{l} i \ne j \\ j = 1 \end{subarray} }^{T} {\varepsilon_{ij} } \frac{{\left( {\pi_{j}^{*} - \pi_{j} } \right)}}{{\pi_{j} }}} \right) $$

where ${P}_{load,i}^{*}$ indicates the peak load after the demand response; ${P}_{load,i}$ represents the demand response to the preload demand; ${\varepsilon }_{ii}$ represents the self-elastic coefficient; $$\sum_{\begin{array}{c}i\ne j\\ j=1\end{array}}^{T}{\varepsilon }_{ij}$$ represents the cross elasticity coefficient; ${\pi }_{i}$ represents the time-of-use price; ${\pi }_{i}^{*}$ represents the TOU price after the demand response; ${\pi }_{j}$ represents the TOU price before the demand response at time *j*; ${\pi }_{j}^{*}$ represents the TOU price after the demand response at time *j.*

5 $$ \left\{ {\begin{array}{*{20}l} {t \in \left[ {T_{f1} ,T_{f2} } \right]} \hfill \\ {P_{MG} = P_{wp} + P_{sp} + P_{stp} } \hfill \\ {\sum\nolimits_{t}^{{T_{f} }} {P_{exc} = \sum\nolimits_{t}^{{T_{f} }} {P_{load,f}^{*} - \sum\nolimits_{t}^{{T_{f} }} {P_{MG} } } } } \hfill \\ {t \in \left[ {T_{p1} ,T_{p2} } \right]} \hfill \\ {P_{MG} = P_{wp} + P_{sp} } \hfill \\ {\sum\nolimits_{t}^{{T_{p} }} {P_{exc} = \sum\nolimits_{t}^{{T_{p} }} {P_{load,p}^{*} - \sum\nolimits_{t}^{{T_{p} }} {P_{MG} } } } } \hfill \\ {t \in \left[ {T_{v1} ,T_{v2} } \right]} \hfill \\ {P_{MG} = P_{wp} + P_{sp} } \hfill \\ {\sum\nolimits_{t}^{{T_{v} }} {P_{exc} = \sum\nolimits_{t}^{{T_{v} }} {P_{load,v}^{*} - \sum\nolimits_{t}^{{T_{v} }} {P_{MG} } + P_{stp} } } } \hfill \\ \end{array} } \right. $$

where ${P}_{MG}$ indicates the microgrid load; ${P}_{wp}$ represents the wind power generation load of microgrid; ${P}_{stp}$ represents the charge and discharge load of microgrid energy storage; ${P}_{exc}$ indicates the load exchange between the microgrid and the external power grid; ${P}_{load,f}^{*}$ indicates the peak load after the demand response; ${P}_{load,p}^{*}$ indicates the normal load after the demand response; ${P}_{load,v}^{*}$ represents the demand response post-valley load.

(2) After the *DR*, the microgrid's power output exceeds the demand from the load, indicated by *P*_*DEG*_ > *P*_*load*_. Furthermore, the power generated by the microgrid is sufficient to support the demand for electricity, including the ability to charge the main power grid or store energy. During periods of high demand, there is no electricity being transferred from the main power grid to the microgrid *MG*, and instead the energy storage system is responsible for supplying power. The interaction power between the *MG* and the large power grid is also zero, indicating that the *MG* is operating independently (in island operation mode). During periods of high demand, *T*_*f*_ utilizes its full energy storage capacity to supply electricity to the major power grid through distributed power supply and micropower sales. During the peak period *T*_*P*_, the *MG* operates autonomously to provide power, and any excess power is stored in the energy storage equipment. During the low-demand phase, the micro grid *MG* operates autonomously to provide power, energy storage, and charging. If there is surplus power, the micro grid can sell it to the main grid to generate cash.

6 $$ P_{load,i}^{*} = P_{load,i} \times \left( {\varepsilon_{ii} \frac{{(\pi_{i}^{*} - \pi_{i} )}}{{\pi_{i} }} + 1 + \sum\limits_{\begin{subarray}{l} i \ne j \\ j = 1 \end{subarray} }^{T} {\varepsilon_{ij} } \frac{{(\pi_{j}^{*} - \pi_{j} )}}{{\pi_{j} }}} \right) $$

7 $$ \left\{ {\begin{array}{*{20}l} {t \in \left[ {T_{f1} ,T_{f2} } \right]} \hfill \\ {P_{MG} { = }P_{wp} + P_{sp} + P_{stp} } \hfill \\ {P_{MG} \ge P_{i,f}^{*} } \hfill \\ {\sum\nolimits_{t}^{T} {P_{exc} = 0} } \hfill \\ {t \in \left[ {T_{p1} ,T_{p2} } \right]} \hfill \\ {P_{MG} = P_{wp} + P_{sp} } \hfill \\ {\sum\nolimits_{t}^{{T_{p} }} {P_{exc} = 0,P_{MG} - P_{i,p}^{*} < E_{es} } } \hfill \\ {\sum\nolimits_{t}^{{T_{p} }} {P_{exc} = P_{MG} - P_{i,p}^{*} - P_{stp} ,P_{MG} - P_{i,p}^{*} > E_{es} } } \hfill \\ {t \in \left[ {T_{v1} ,T_{v2} } \right]} \hfill \\ {P_{MG} = P_{wp} + P_{sp} - P_{es} } \hfill \\ {\sum\nolimits_{t}^{{T_{v} }} {P_{exc} = \sum\nolimits_{t}^{{T_{v} }} {P_{MG} - } P_{i,v}^{*} } } \hfill \\ \end{array} } \right. $$

where ${P}_{i,f}^{*}$ indicates the load after the demand response in peak hours; ${P}_{i,p}^{*}$ represents the user load after demand response in normal peak time; ${P}_{i,v}^{*}$ represents the user load after the demand response in the low moment.

Equations (4) and (6) both describe the load after the demand response, but they each represent different working conditions. Equation (4) is suitable for the demand reduction scenario where the output power is lower than the actual load, while Eq. (6) is for the demand supplement situation where the output power is higher than the actual load. It is further explained and distinguished by Eqs. (5) and (7), which respectively specify the processing logic and parameter changes in the corresponding scenario. In addition, in the DR Model, the self-elasticity coefficient is greater than 1, indicating that the TOU price after the demand response increases with the increase of the load during the peak period after the demand response. The self-elasticity coefficient is less than 1, indicating that the TOU price after the demand response decreases with the decrease of the load during the peak period after the demand response. The positive cross elasticity coefficient indicates that the TOU price before the demand response at time *j* is positively correlated with the TOU price after the demand response at time *j*. The negative cross elasticity coefficient indicates that the TOU price before the demand response at time *j* is negatively correlated with the TOU price after the demand response at time *j*.

### Optimize objective function before optimizing price

The interruptible load policy is an economic contract (agreement) signed by the power company and the user in advance. When the system is at its peak and in an emergency state, the user interrupts or reduces. The load is in accordance with the terms of the contract, and the power company provides the user with specific economic compensation. For power enterprises, when the power shortage, cannot meet the needs of all users. In order to ensure the overall power balance, it is necessary to interrupt part of the load. In order to induce users to respond positively during the peak period, the power supplier must provide reasonable incentive mechanism and compensation mechanism. For the user, in the environment of the power market, the price of electricity changes in real time. In case of power shortage, the price of electricity may be higher than the cost of the user's power shortage. At this time, the user can consciously choose to cut off electricity through the interruptible load contract, and thus obtain certain economic compensation. Therefore, in the implementation of interruptible load contract, the income balance determines whether the interruptible load policy is feasible.

When it comes to managing the distribution of power grid load, users prioritize electricity price over incentive programs. Power companies can achieve the transfer of load in a controllable manner by implementing a flexible TOU pricing structure. An optimized TOU pricing model can effectively encourage customers to actively engage in load regulation, ensuring efficient management of load peaks and valleys and appropriate allocation of resources.

(1) Maximizing user delight to the fullest extent

In the above equation, ${R}_{exp}$ represents the level of satisfaction with electricity spending, ${R}_{com}$ represents the degree of comfort with energy usage, $\pi$ represents the amount of electricity fee paid by users. Normalizing each indicator can be obtained:

8 $$ \begin{aligned} \max F_{1} & = \alpha R{}_{\exp } + \beta R{}_{com} \\ & = \alpha \left( {1 - \frac{{\sum\nolimits_{t = 1}^{T} {\left| {\pi_{t}^{*} P_{t}^{*} - \pi_{t} P_{t} } \right|} }}{{\sum\nolimits_{t = 1}^{T} {\pi_{t} P_{t} } }}} \right) + \beta \left( {1 - \frac{{\sum\nolimits_{t = 1}^{T} {\left| {P_{t} - P_{t}^{*} } \right|} }}{{\sum\nolimits_{t = 1}^{T} {P_{t} } }}} \right) \\ \end{aligned} $$

9 $$ \alpha + \beta = 1 $$

(2) Cost minimization of microgrid

To optimize the cost of the micro grid, the first thing is to ensure the safety and stability of the power grid operation, and then consider: the photovoltaic operation cost of the micro grid in the dispatching cycle; Power purchase cost of external network of micro grid; The operating costs of wind power; The running costs of energy storage. In formula 10, *C*_*wp*_ is the photovoltaic operation cost of the microgrid in the dispatching cycle; *C*_*buy*_ is the power purchase cost of the micro-grid to the external network; *C*_*sp*_ is the operating cost of wind power in the micro-grid during the dispatching cycle; *C*_*stp*_ is the operating cost of energy storage in the micro-grid during the dispatching cycle. *R*_*wp*_, *R*_*sp,*_* R*_*stp*_ is the operation and maintenance cost of light, wind and energy storage units in the micro-grid during the dispatching cycle.

10 $$ \begin{aligned} & \min F_{2} = C_{wp} + C_{sp} + C_{stp} + C_{buy} \\ & \left\{ \begin{gathered} C_{wp} = \sum\nolimits_{t}^{T} {P_{wp} \times R_{wp} } \hfill \\ C_{sp} = \sum\nolimits_{t}^{T} {P_{{{\text{sp}}}} \times R_{sp} } \hfill \\ C_{stp} = \sum\nolimits_{t}^{T} {P_{stp} \times R_{sp} } \hfill \\ C_{buy} = \sum\nolimits_{t}^{T} {\Delta P_{exc} \times \pi_{i} } \hfill \\ \end{gathered} \right. \\ \end{aligned} $$

### Consider the objective function of user satisfaction

Considering the criticality of the self-organization of the microgrid, the user satisfaction is reformed, and the overall blackout loss mainly complements the overall profit of the original users and the microgrid. At this time, the target function is adjusted to:

(1) Improved user satisfaction model

In the above equation, *R*_*sl*_ is the user subsidy to compensate for the power shortage loss after the demand response. *R*_*loss*_ is the user satisfaction with an interruptible load; ${\partial }_{i}$ indicates that the higher the impact of the interruptible load policy on the user on the power load of the time period, the lower the user satisfaction during the interruption.

11 $$ P_{load,f}^{*} = P_{load,f} \times \left( {1 + \varepsilon_{ff} \frac{{(\pi_{f}^{*} - \pi_{f} ) + R_{sl} + R_{tl} }}{{\pi_{f} }} + \sum\limits_{\begin{subarray}{l} i \ne j \\ j = 1 \end{subarray} }^{T} {\varepsilon_{ij} } \frac{{(\pi_{j}^{*} - \pi_{j} ) + R_{tl} }}{{\pi_{j} }}} \right) $$

12 $$ R_{{{\text{loss}}}} = 1 - \partial_{i} \frac{{\Delta P_{load} \times R_{sl} }}{{P_{load} \pi }} $$

13 $$ \begin{aligned} \max F_{1} & = \alpha R{}_{\exp } + \beta R{}_{com} + \gamma R{}_{{{\text{loss}}}} \\ & = \alpha \left( {1 - \frac{{\sum\nolimits_{t = 1}^{T} {\left| {\pi_{t}^{*} P_{t}^{*} - \pi_{t} P_{t} } \right|} }}{{\sum\nolimits_{t = 1}^{T} {\pi_{t} P_{t} } }}} \right) + \beta \left( {1 - \frac{{\sum\nolimits_{t = 1}^{T} {\left| {P_{t} - P_{t}^{*} } \right|} }}{{\sum\nolimits_{t = 1}^{T} {P_{t} } }}} \right) + \gamma \left( {1 - \partial_{i} \frac{{\Delta P_{load} \times R_{sl} }}{{P_{load} \pi }}} \right) \\ \end{aligned} $$

14 $$ \alpha + \beta + \gamma = 1 $$

(2) Dynamic and variable cost of microgrid operation

In the above equation, *C*_*buy*_ is the power purchase cost of the micro-grid to the external network; *C*_*sl*_ is the interruption load subsidy to compensate the user for the power loss; *C*_*tl*_ is the transferable load subsidy for the user; $\Delta {P}_{exc}$ indicates the power interaction between microgrid and external network; When the microgrid buys electricity online, ${\pi }_{i}$ means the time-sharing electricity price, and when the microgrid sells electricity online; $\Delta {P}_{load}$ is the overall interruptible load volume; *R*_*sl*_ Is the unit interruptible load subsidy; *P*_*fv,fp,pv*_ is the load transfer from peak period to trough period, peak period to flat peak period, and flat peak period to trough period; *R*_*fv,fp,pv*_ is the unit can transfer the load subsidy.

15 $$ \begin{aligned} & \min F_{2} = C_{wp} + C_{sp} + C_{stp} + C_{buy} + C_{sl} + C_{tl} \\ & \left\{ \begin{gathered} C_{wp} = \sum\nolimits_{t}^{T} {P_{wp} \times R_{wp} } \hfill \\ C_{sp} = \sum\nolimits_{t}^{T} {P_{{{\text{sp}}}} \times R_{sp} } \hfill \\ C_{stp} = \sum\nolimits_{t}^{T} {P_{stp} \times R_{sp} } \hfill \\ C_{buy} = \sum\nolimits_{t}^{T} {\Delta P_{exc} \times \pi_{i} } \hfill \\ C_{sl} = \sum\nolimits_{t}^{T} {\Delta P_{load} \times R_{sl} } \hfill \\ C_{tl} = \sum\nolimits_{t}^{T} {P_{fv,fp,pv} \times R_{fv,fp,pv} } \hfill \\ \end{gathered} \right. \\ \end{aligned} $$

### Constraint condition

(1) Overall operation power balance of microgrid

16 $$ P_{load} = P_{wp} + P_{sp} + P_{stp} + P_{exc} $$

In formula, *P*_*load*_ is the user power load in the micro-grid; *P*_*wp*_ is the wind power generation in the micro-grid; *P*_*sp*_ is the photovoltaic power generation in the micro-grid; *P*_*stp*_ is the energy storage charge and discharge load in the micro-grid; *P*_*exc*_ is the interaction load of micro-grid and external network.

(2) Interactive power constraint between microgrid and external network

To ensure the safe and reliable operation of grid and micro grid interaction, the interaction power of grid and external network should be within the prescribed allowable limit.

17 $$ P_{exc,\min } \le P_{exc} \le P_{exc,\max } $$

In formula, *P*_*exc,min*_, *P*_*exc,max*_ is the minimum and maximum value of the interaction between the *MG* and the external network, respectively.

(3) Energy storage battery constraints in microgrid

18 $$ \left\{ {\begin{array}{*{20}l} {0 \le P_{n}^{es} \le P_{n,\max }^{es} } \hfill \\ {SOC\left( {t_{\min } } \right) \le SOC\left( t \right) \le SOC\left( {t_{\max } } \right)} \hfill \\ {P_{start}^{es} = P_{end}^{es} } \hfill \\ \end{array} } \right. $$

In formula, *SOC*(*t*) is the charge and discharge state at time t of the energy storage equipment in the microgrid; ${P}_{start}^{es}$ and ${P}_{end}^{es}$ indicate that the charge state before and after the charge and discharge of the energy storage equipment should be consistent, that is, the energy storage equipment itself has no power loss, and shall be restored to the original power after one cycle of operation.

(4) User load peak constraint

Notably, new peaks are not allowed during valley periods after demand response.

19 $$ \mathop {\max }\limits_{{DR\left( {t \in t_{g} } \right)}} P_{Load} \le \mathop {\max }\limits_{{DR\left( {t \in t_{f} } \right)}} P_{Load} $$

In formula, $\underset{\mathit{DR}(t\in {t}_{g})}{\text{max}}{P}_{load}$ is the maximum power load in the trough period after the *DR,* $\underset{\mathit{DR}(t\in {t}_{f})}{\text{max}}{P}_{load}$ represents the maximum load during peak hours after *DR*.

### Optimization algorithm

As an effective organization form of distributed power supply and a powerful supplement to large power grid, microgrid has become one of the development directions of smart power grid in the world. The operation cost of micro grid can be saved and the price of electricity can be reduced effectively by rationally arranging the working state of micro power supply and load management. Aiming at the double-objective optimization problem of the user side and the power supply side of the micro grid in the power market, this study adopts the double-objective optimization algorithm to find the optimal solution of the price problem, that is, the optimal price when the two sides reach the game equilibrium. This paper is the dual-target optimization problem of the microgrid user side and the power supply side in the power market. In view of the dual-target optimization problem, the current scholars believe that the best solution is to find the optimal solution to the problem, that is, the optimal electricity price when the two sides reach the game equilibrium. Therefore, this paper solves it with the chaotic particle group algorithm. The objective function is specially set to maximize the maximum user satisfaction and the reciprocal cost of the power supply enterprise.

**Figure 4:**
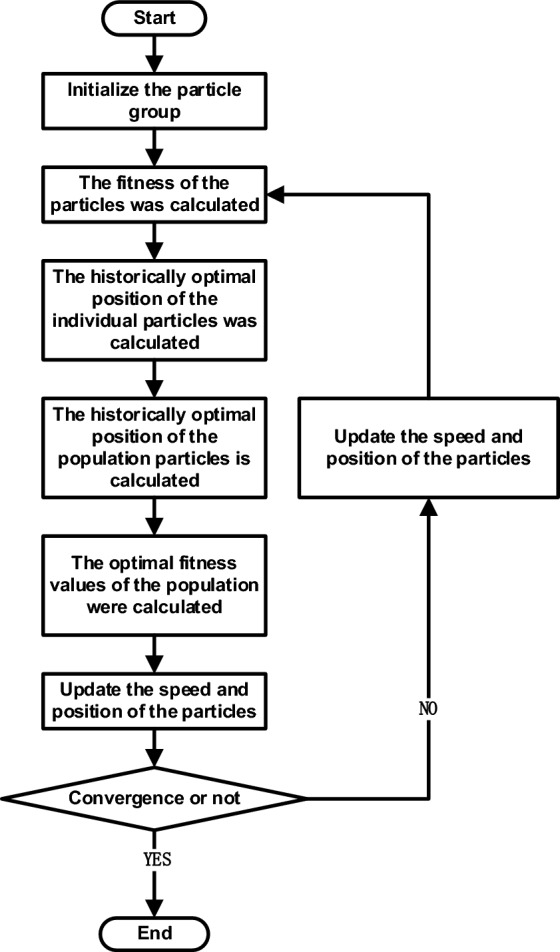
The flow chart of *PSO.*

The optimization model was solved using the CPSO in Matrix Laboratory (MATLAB) software to obtain the user peak and flat valley time-sharing electricity price. MATLAB is MathWorks for the development of algorithms, data visualization, data analysis and numerical calculation of advanced technical computing language and interactive environment of business mathematics software. MATLAB has the functions of numerical analysis, numerical and symbolic calculation, engineering and scientific drawing, digital image processing, finance and financial engineering, etc., providing a comprehensive solution for many scientific fields. Standard PSO through simulation of bird foraging random solution, easy into local optimal situation. The overall flow chart of the standard particle algorithm is shown in Fig. 4.

(1) Principle of particle swarm optimization (PSO)

In a D-dimensional space, we assume that there are *N* particles, and these particles form a population. In the PSO, the *i*th particle represents and its flight speed is denoted by *V*_*best*_, where $V_{best} = \{ v_{i1} ,v_{i2} ,v_{i1} ,\;v_{iD} \}$. The individual extremum found by the ith particle:$P_{best} = \{ p_{i1} ,p_{i2} ,p_{i1} ,p_{iD} \}$. The optimal solution searched by the whole population is the global extremum:$G_{best} = \{ {\kern 1pt} g_{i1} ,\;g_{i2} ,{\kern 1pt} g_{i1} ,{\kern 1pt} g_{iD} \}$, *i* is taken as [1, *N*]. The particle will then update its velocity and position according to Eqs. (20) and (21)^33^:

20 $$ v_{id}^{k + 1} = wv_{id}^{k} + c_{1} r_{1} \left( {p_{id} - x_{id}^{k} } \right) + c_{2} r_{2} \left( {G_{id} - x_{id}^{k} } \right) $$

21 $$ x_{id}^{k + 1} = x_{id}^{k} + v_{id}^{k + 1} $$

where *w* is inertia weight; $c_{1}$ is the acceleration of self-learning; $c_{2}$ is the acceleration of social learning; $r_{1}$ and $r_{2}$ are random numbers ranging from 0 to 1.

(2) Optimization of chaotic particles

Chaotic particles use the characteristics of their own motion to make the population jump out of the local optimum and reach the global optimum. In this paper, the Logistic map chaotic sequence is used, which can be updated and transformed repeatedly according to the iterative equation to enhance the randomness of the algorithm^34^.

22 $$ x_{n + 1} = \mu x_{n} (1 - x_{n} ) $$

where *n* is the current iteration number. When the value of *μ* is close to 4, complete chaotic particles are generated, and the value generated by iteration is more suitable for combinatorial optimization^34^.

23 $$ x_{n + 1} = 4x_{n} (1 - x_{n} ) $$

The motion characteristics of chaotic particles can help the population to find the optimal solution. The core of the search is to generate a set of variables, and the number of the variables is the same as that of the optimization variables. This variable is used as a carrier and an optimization variable is introduced to make it behave as a chaotic state. Secondly, it traversed its motion range to make it meet the value of the optimization variable. Finally, it searched through the chaotic variable. The basic steps are as follows:

(1) Generate n chaotic variables $Z_{i} = ({\kern 1pt} Z_{i - 1} ,\;Z_{i - 2} ,\; \ldots ,\;{\kern 1pt} Z_{i - n} )$ with different trajectories according to Eq. (23)

(2) The individual components of $Z_{i}$ are carried to the chaotic disturbance range $[ - \beta ,\beta ]$, disturbance quantity $\Delta x = ({\kern 1pt} \Delta x_{1} ,\;{\kern 1pt} \Delta x_{2} ,\;{\kern 1pt} \ldots ,\;{\kern 1pt} \Delta x_{n} )$, and new particles are generated, where

24 $$ \Delta x_{j} = - \beta + 2\beta Z_{ij} $$

25 $$ x_{new} = g_{best} k + \Delta x $$

(3) Recalculate the individual extreme value of the particle, and update the position information of the particle if the extreme value is less than the global extreme value.

**Figure 5:**
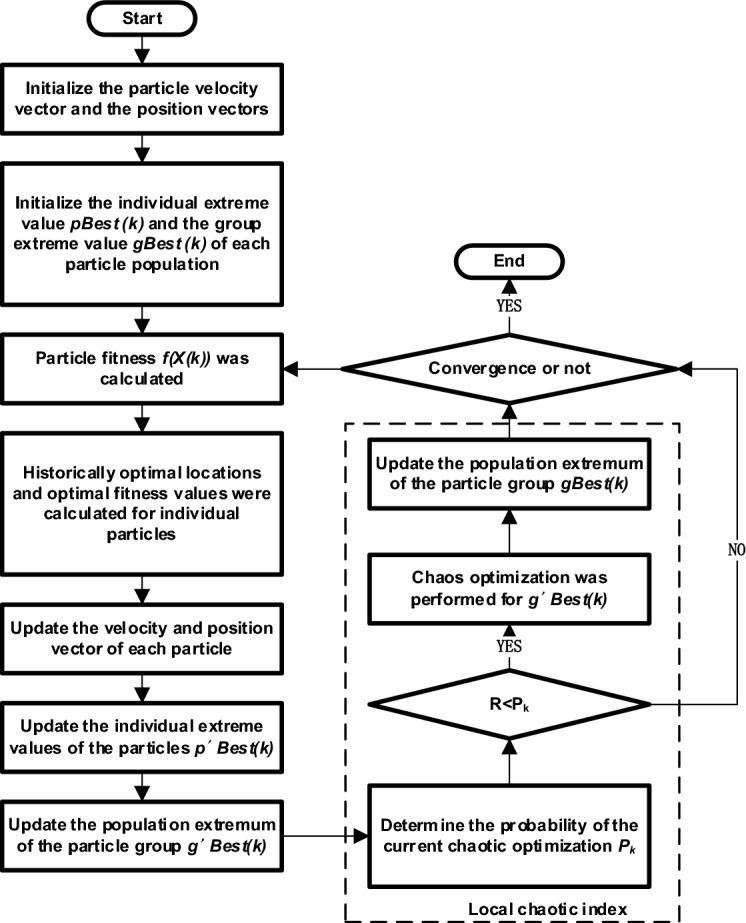
The flow chart of CPSO.

Compared with the traditional particle swarm optimization algorithm, chaotic particle swarm optimization algorithm introduces chaotic process into the particle swarm optimization algorithm to improve the breadth and depth of its optimization, obtain higher quality hyperparametric solutions, and improve the regression prediction effect of the mathematical model in this study. In other words, in the CPSO, the initial position and velocity of the particle group are reset through the chaotic sequence; second, in the CPSO, the chaotic disturbance is introduced adjacent to the optimal solution position, and the optimal solution is quickly found. The flow chart of chaotic particle swarm optimization algorithm as shown in Fig. 5.

**Figure 6:**
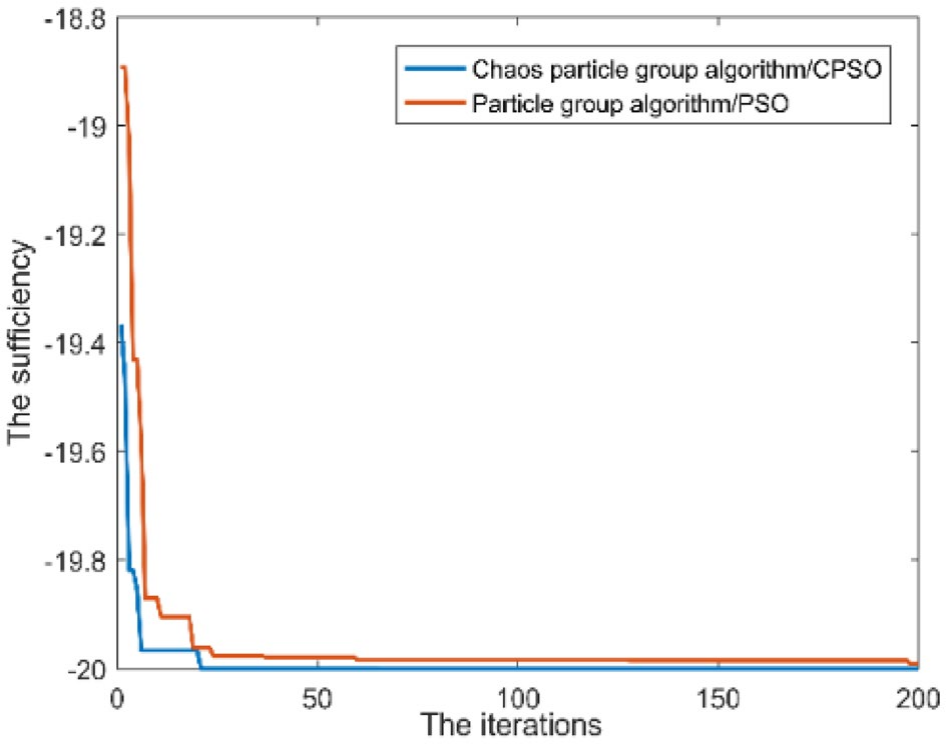
Comparison of optimization algorithms of PSO and CPSO.

To more reflect the difference between chaotic particle group algorithm and particle group algorithm, the paper introduces the “*Rastrigin function*”, which is known to be at a global minimum at (0,0). The results performed based on the MATLAB data programming software are shown in Fig. 6. The chaotic particle group algorithm runs faster and has a more significant effect. Through the Rastrigin function, it can be reflected laterally that chaotic particle swarm optimization algorithm runs faster and has a more significant effect in the dual-objective solving process of demand response.

## Results and discussion

To better reflect the real reliability of the problems proposed in this paper, this paper uses the daily load control data of residents in microgrid for analysis. Among them, T = 24, ∆t = 1. Among them, the relevant parameters of wind and solar storage in the microgrid are from literature^35,36^. The microgrid in the active distribution network is mainly composed of Distributed Generation (DG) units, mainly including renewable energy power generation (PV, WT) and ES systems.

To verify the superiority of the study scheme, two microgrid load optimization control schemes are analyzed and compared.

1. Scenario1: Develop strategies for microgrid time-sharing prices considering only price *DR.*
2. Scenario2: Micro-grid user load optimization scheduling strategy considering integrated *DR.*

In the calculation process, to reflect the limitation of model construction, the overall correspondence of users in microgrid is proposed, as shown in Eq. (26).

26 $$ \mu_{load} = \frac{{\sum\nolimits_{t}^{T} {\Delta P_{load} } }}{{P_{load} }} $$

In formula, ${\mu }_{load}$ is the overall user response in the *MG*; ${P}_{load}$ is the multitype user demand response preload of the *MG*; ${\Delta P}_{load}$ is the poor load before and after the DR of the *MG*.

### Scenario 1: Develop strategies for microgrid time-sharing prices considering only price demand response

The interaction limit between microgrid and external power grid is [− 200 KW, 200 KW], the rated capacity of energy storage equipment is to 90 kW, the upper and lower limits of state of charge are to 1.0 and 0.2, the charge/discharge power efficiency is to 90%, and the initial state of charge is to 0.2. Set the initial state of energy storage to full power, which is $SOC\left({t}_{1}\right)$ = 0.9.

**Figure 7:**
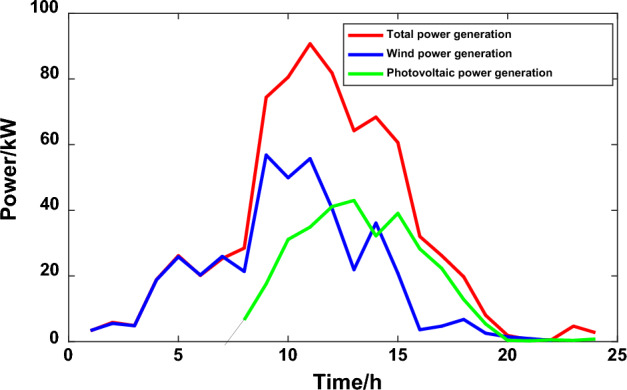
Output curve of MG distributed power generation.

**Figure 8:**
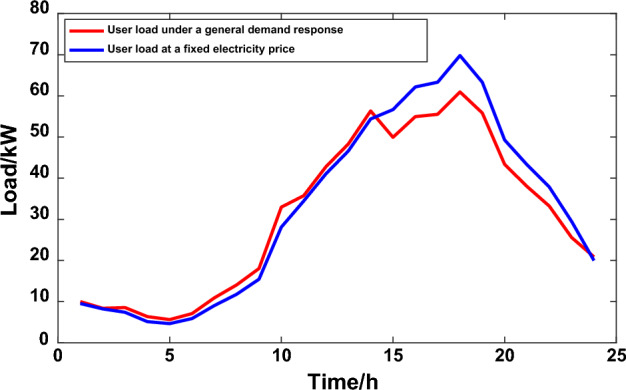
User load in the microgrid.

The starting fixed electricity price of the power grid is 0.55 yuan/kW·h, micro network electricity sales price is 0.45 yuan/kW·h. The user satisfaction parameters were all 0.5. When formulating the law based on the chaotic particle group algorithm and the traditional peak and valley time sharing price, the whole day is divided into peak, flat and valley periods, keeping the normal electricity price the same as the fixed electricity price, and the peak and valley periods are 0.85 yuan/kW·h and 0.26 yuan/kW·h, respectively. The electricity price division period is included in Table 1, the interruptible load accounts for 15%, and the terminal load subsidy is 0.1 yuan/kW·h. The operation and maintenance cost of photovoltaic units in the micro-grid is 0.0096 yuan/kW·h, the wind power unit is 0.00296 yuan/kW·h, and the operation and maintenance cost of energy storage equipment is 0.005 yuan/kW·h. After calculation, the overall electricity payment of users in scenario 1 is 500.45 yuan, and the user interruption load ratio is 4.23%. In scenario 1, the output curve of MG distributed power generation and the user load curve in microgrid are shown in Figs. 7 and 8 respectively.

**Table 1:** Analysis of various types of microgrid data under scenario 1.

Time interval	Period	TOU Price	User satisfaction	User responsiveness	Load peak and valley difference/KW	Microgrid electricity cost/ten thousand yuan
Peak period	15:00–23:00	0.85	0.5327	0.0435	55.835	111.51
Flat period	10:00–15:00	0.55				
	23:00–02:00					
Trough period	2:00–10:00	0.26

### Scenario 2: Micro-grid user load optimization scheduling strategy considering the integrated demand response

**Figure 9:**
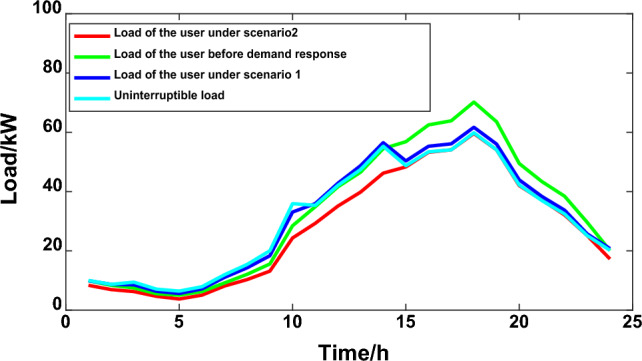
User load under Scenario 2.

In a simulation analysis of the microgrid multi-objective optimization scheduling model based on demand-side management using the chaotic particle group algorithm, the optimization algorithm was set to iterations at 100, cross probability pc = 0.9, and variant probability pm = 0.1. The improved user satisfaction and the total operating cost of the microgrid are set as a multi-objective optimization problem. Combined with the transferable load characteristics in the microgrid, the load transfer amount to peak period is 8 kW, the load to trough transfer coefficient is 10 kW during peak period, the load transfer coefficient during peak period is 6 kW, the transfer load compensation rate is 0.1 yuan/kW·h, and the interruption load subsidy is 0.15 yuan/kW·h. The user load and time-sharing electricity price parameters under scenario 2 are shown in Fig. 9 and Table 2.

**Table 2:** Load data after optimization of TOU pricing strategy.

Time interval	Period	TOU price	User satisfaction	User responsiveness	Peak-valley difference/KW	Microgrid electricity cost/ten thousand yuan
Peak period	15:00–23:00	1.3646	0.6023	0.05535	53.26	98.5348
Flat period	10:00–15:00	0.595				
	23:00–02:00					
Trough period	2:00–10:00	0.2757				

**Figure 10:**
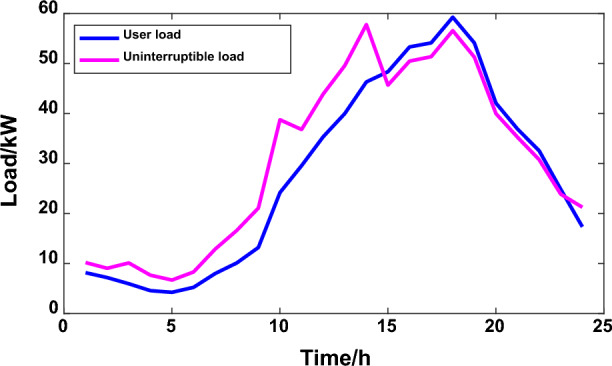
User load under a traditional demand response.

To highlight the optimization scheme of the model microgrid load control proposed in this article, the score electricity price is once again brought into the traditional *DR*, and the results are shown in Fig. 10. Under the load control measures under the traditional peak and valley electricity price model, the electricity consumption level basically only meets the minimum demand, and cannot meet the comfort requirements of user electricity consumption, resulting in low user satisfaction.

### Comparison of different scenarios

**Figure 11:**
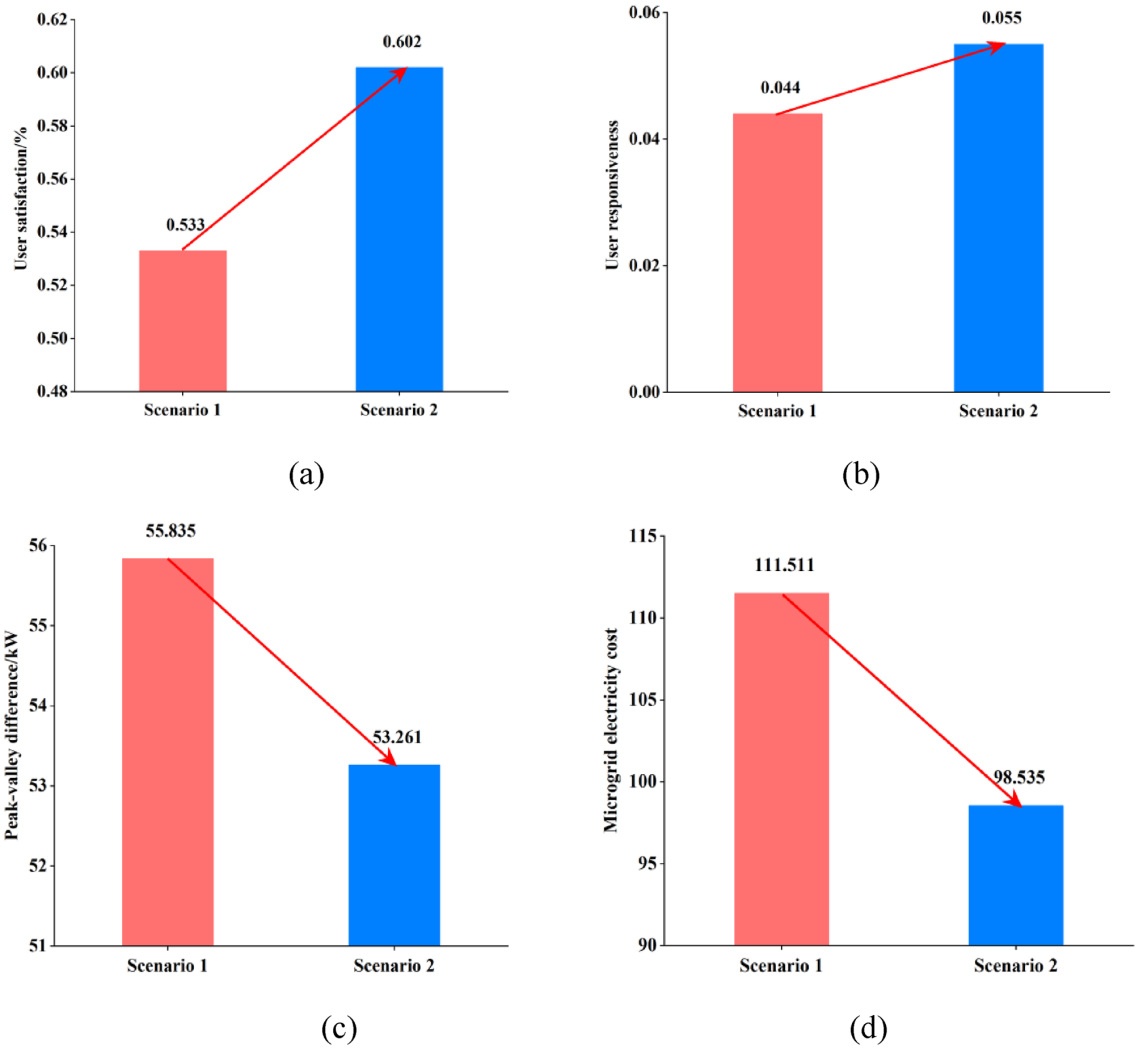
Comparative benefits of microgrid in two scenarios.

According to Table 3 and Fig. 11, comparing the microgrid load optimization scheduling results under different demand response strategies of scenario 1 and scenario 2 with the optimal solution of the overall user satisfaction, only the microgrid user load curve under the influence of price demand response is considered, the load peak and valley difference is smaller and the overall user satisfaction is lower. Using the optimal dispatching of microgrid under the influence of the combination of TOU price and incentive policy, the overall user satisfaction increased by 9.51%, the overall operation cost of microgrid power supplier decreased by 12.975/ten thousand yuan, the peak valley difference decreased by 4.61%, and the user demand response increased by 27.24%. Therefore, the strategy proposed in this paper enhances the peak and valley elimination ability of active distribution network, smoothers the power demand curve, and further improves the load optimization management and control.

**Table 3:** Comparison of comprehensive benefits of microgrid under two scenarios.

Scenes	User satisfaction	User responsiveness	Peak-valley difference/kW	Microgrid electricity cost//ten thousand yuan
Scenario 1	0.533	0.044	55.835	111.510
Scenario 2	0.602	0.055	53.260	98.535

### Comparison of similar algorithms

CPSO algorithm is a collaborative optimization method based on PSO, which seeks the global optimal solution through the cooperation and information exchange between particles. In multi-objective optimization, there are many conflicting objectives, and it is impossible to find a unique optimal solution simply. Pareto-front is the core concept to solve this problem. Pareto-front refers to the set of all non-inferior solutions in multi-objective optimization, where the improvement of one objective means the deterioration of at least one other objective. By using CPSO algorithm to achieve simultaneous optimization of both price and incentive, a series of non-inferior solutions can be found to explore the tradeoff between price and incentive. Such a solution set can provide decision-makers with a more comprehensive choice space, enabling them to choose the most suitable solution according to their specific needs and preferences. In conclusion, by using the CPSO algorithm and based on the concepts of the Pareto-front, this study achieves the simultaneous optimization of both price and incentive objectives, providing a more comprehensive consideration framework for decision makers, which is of great significance for weighing multiple objectives and choosing the best decision.

Studies have shown that the computational complexity of an algorithm is an important index to measure the quality of an algorithm^37^. The smaller the computational complexity of the algorithm, the shorter the running time of the algorithm. The computing configuration used in this study is: running on 64-bit Win10 operating system, equipped with Intel Core i7-9750h processor, equipped with NVIDIA GeForce RTX 2060 6 GB graphics card, and has 16 GB memory. The comparison results of different algorithms are shown in Table 4

**Table 4:** Comparison of different algorithms.

Scenes	User satisfaction	User responsiveness	Peak-valley difference/kW	Microgrid electricity cost//ten thousand yuan	Running time (s)
Scenario 1 (PSO)	0.533	0.044	55.835	111.510	106.52
Scenario 2 (CPSO)	0.602	0.055	53.260	98.53	92.67

According to the data in Table 4, CPSO algorithm has higher user satisfaction and user convenience than PSO algorithm. At the same time, the thermoelectric difference, the electricity cost of the microgrid and the running time of the algorithm are lower than that of the PSO algorithm.

Common multi-objective optimization algorithms include Multi-objective genetic algorithm (MOGA), Multi-objective differential evolution (MODE), Non-dominated sorting genetic algorithm (NSGA) and Multi-objective ant colony optimization (MOACO) , etc. This study uses these commonly used optimization methods to optimize the problem of this study, and the results are shown in Table 5.

**Table 5:** Comparison between different optimization algorithms.

Methods	User satisfaction	User responsiveness	Peak-valley difference/kW	Microgrid electricity cost//ten thousand yuan	Running time (s)
MOGA	0.512	0.039	57.135	115.510	110.65
MODE	0.526	0.041	56.123	113.682	108.79
PSO	0.533	0.044	55.835	111.510	106.52
MOACO	0.546	0.049	54.321	105.863	99.35
NSGA	0.578	0.052	53.879	103.467	95.67
CPSO (this study)	0.602	0.055	53.260	98.535	92.67

It can be seen from Table 5 that compared with other multi-objective optimization methods, the CPSO method adopted in this study has better performance in terms of user satisfaction and user convenience, and lower performance in terms of thermoelectric difference, microgrid electricity cost and algorithm running time.

## Conclusion

The original microgrid load control model based on demand response lacks the incentive demand response factors, the overall user satisfaction is low, the low demand response degree, the time-sharing electricity price of the formulated peak and valley filling capacity is weak, and the peak and valley difference of the load curve is high.

In view of the above problems, this paper further optimizes the flexible load control strategy and proposes a two-objective optimization model based on price and incentive. Meanwhile, the model is solved using an improved chaotic particle group algorithm. Finally, the microgrid load data were selected for simulation analysis. The simulation results showed that the comprehensive demand response of flexible control model proposed increased the overall satisfaction of users by 9.51%, the overall operating cost of microgrid suppliers decreased by 12.975/ ten thousand yuan, the peak valley difference decreased by 4.61%, and the user demand response increased by 27.24%. The model effectively improves the overall profit of the supply side of the microgrid, improves the user satisfaction, and maximizes the linkage benefits of the supply and demand of the micro grid. The model effectively improves the maximum profit of microgrid, improves the position of power users in the power market, effectively reduces the phenomenon of distributed power supply in microgrid, and realizes the supply–demand matching of the overall load of microgrid (Supplementary information).

### Supplementary Information

Supplementary Information.

## Data Availability

The datasets used and analyzed during the current study are available from the corresponding author on reasonable request.
