# Supplementary material for: Optimization scheduling of microgrid comprehensive demand response load considering user satisfaction
Source: Sci Rep. 2024 Jul 11;14:16034. doi: 10.1038/s41598-024-66492-1 (PMC11239861; doi:10.1038/s41598-024-66492-1)
Supplement: Supplementary file 1 — Supplementary Information. [file 41598_2024_66492_MOESM1_ESM.docx]

**Appendix I**

| DR | Demand response |
| --- | --- |
| ES | Energy storage |
| DES | distributed energy storage |
| IDR | Incentive demand response |
| DLC | Direct Load Control |
| IL | Interruptible Load |
| DSB | Demand Side Bidding |
| EDR | Emergency Demand Response |
| PDR | Price based demand response |
| TOU | Time of use tariff |
| RTP | Real Time Pricing |
| CPP | Critical Peak Pricing |
| WP | wind power |
| PV | photovoltaic power |
| DG | distributed generation |
| DEG | distributed energy generation |
| CPSO | chaos theory-based particle group optimization |
| PSO | particle group algorithm |
| MG | micro grid |
